# Site-specific chemical conjugation of human Fas ligand extracellular domain using *trans*-cyclooctene – methyltetrazine reactions

**DOI:** 10.1186/s12896-017-0381-2

**Published:** 2017-07-03

**Authors:** Michiro Muraki, Kiyonori Hirota

**Affiliations:** Biomedical Research Institute, National Institute of Advanced Industrial Science and Technology (AIST), Central 6, 1-1-1 Higashi, Tsukuba, Ibaraki 305-8566 Japan

**Keywords:** Human Fas ligand, Extracellular domain, Site-specific conjugation, *trans*-Cyclooctene, Methyltetrazine, Fluorochrome, Functional protein, Receptor-binding activity

## Abstract

**Background:**

Fas ligand plays a key role in the human immune system as a major cell death inducing protein. The extracellular domain of human Fas ligand (hFasLECD) triggers apoptosis of malignant cells, and therefore is expected to have substantial potentials in medical biotechnology. However, the current application of this protein to clinical medicine is hampered by a shortage of the benefits relative to the drawbacks including the side-effects in systemic administration. Effective procedures for the engineering of the protein by attaching useful additional functions are required to overcome the problem.

**Results:**

A procedure for the site-specific chemical conjugation of hFasLECD with a fluorochrome and functional proteins was devised using an inverse-electron-demand Diels-Alder reaction between *trans*-cyclooctene group and methyltetrazine group. The conjugations in the present study were attained by using much less molar excess amounts of the compounds to be attached as compared with the conventional chemical modification reactions using maleimide derivatives in the previous study. The isolated conjugates of hFasLECD with sulfo-Cy3, avidin and rabbit IgG Fab’ domain presented the functional and the structural integrities of the attached molecules without impairing the specific binding activity toward human Fas receptor extracellular domain.

**Conclusions:**

The present study provided a new fundamental strategy for the production of the engineered hFasLECDs with additional beneficial functions, which will lead to the developments of the improved diagnostic systems and the effective treatment methods of serious diseases by using this protein as a component of novel molecular tools.

**Electronic supplementary material:**

The online version of this article (doi:10.1186/s12896-017-0381-2) contains supplementary material, which is available to authorized users.

## Background

Fas ligand (FasL) plays a key role in preventing many serious diseases in the human immune system as a major cell death inducing protein [1, 2]. The extracellular domain of human Fas ligand (hFasLECD) binds to human Fas receptor (hFasR) on the surface membrane of malignant cells and triggers apoptosis of the target cells. Therefore, it is expected that hFasLECD has substantial promising potentials in the field of medical biotechnology [3, 4]. The intravenous administration of a large amount of hFasLECD produced in *Pichia pastoris* caused a serious liver injury by acute hepatitis. However, the specific activity of the hFasLECD sample was at least 20 times higher than an anti-mouse FasR agonistic monoclonal antibody, Jo2, in inducing apoptosis against FasR overexpressing mouse cells, and showed much less toxicity with regard to the liver failure *in vivo* [5]. To overcome the above mentioned problem, numerous studies for delivering the protein specifically toward the target cells have been made by exploiting the gene-fusion technology using the genes of single chain variable fragments of the cell-surface antigen recognizing antibodies and the extracellular domains of cytokines as the fusion components [6–10]. On the other hand, the administration of many cytotoxic drugs, including the ones in clinical uses, is known to significantly affect the number of cell-surface hFasR, which determines the susceptibility to apoptosis execution by hFasL [11–14]. Accordingly, engineered molecules, including antagonistic monoclonal antibodies against the extracellular domain of hFasR (hFasRECD) such as ZB4, have been also developed as useful molecular tools for the detection of cell-surface hFasR [15, 16].

Site-specific chemical conjugation utilizing a reactive tag residue to install chemical groups by covalent additions is another potent technology for engineering proteins to attach new functionalities, which are not available in the original molecules [17, 18]. In previous studies, one of the authors has developed an hFasLECD derivative containing a reactive cysteine residue in its N-terminal tag sequence [19], and prepared a functional fluorescent derivative as a prototype engineered molecule by direct chemical modification of the cysteine residue using a large excess molar amount of fluorescein 5-maleimide, without impairing original hFasRECD binding activity [20]. However, the free thiol groups in the cysteine residues tend to lose the reactivity by oxidative disulfide-bridges formation, and the maleimide-groups in the fluorochrome labeling reagents can be readily inactivated by hydrolysis, under aqueous buffer conditions of physiological pH. Recently, a powerful means for chemical conjugations, which employs an inverse-electron-demand Diels-Alder reaction between *trans*-cyclooctene (TCO) group and methyltetrazine (MTZ) group, has been developed as an efficient tool in the field of bioorthogonal click chemistry [21], and a variety of relevant chemical reagents became commercially available. TCO and MTZ groups are fairly stable in physiological aqueous buffer solutions, and the conjugation reaction between them can proceed with exceptionally fast kinetics and high selectivity [22, 23]. This makes the reaction attractive for the applications in which only a limiting amount of molecules to be conjugated are generally available, such as the cases using expensive low molecular-weight compounds or precious functional proteins. However, in spite of its potential usefulness, the behaviors in actual conjugation events are not always well documented yet.

In this study, in order to seek the possibility of extending the functionalities to be attached using a less molar excess amount of modification reagents, site-specific chemical conjugations of a hFasLECD derivative were investigated using the TCO - MTZ cycloaddition reaction. Sulfo-Cy3 fluorochrome derivatives, an avidin derivative and a rabbit Fab’ domain derivative were each employed as a representative molecule of low molecular-weight compounds, protein molecules modified with multiple reactive groups and protein molecules containing a single reactive group, respectively. The isolated samples of the conjugates were characterized for their functional and structural integrities of both components in the conjugates using spectroscopic measurements and detection of complex formation with human Fas receptor extracellular domain-human IgG_1_ Fc domain fusion protein (hFasRECD-Fc) as well as each individual specific binder.

## Results

### Conjugation design and procedures

**Fig. 1:**
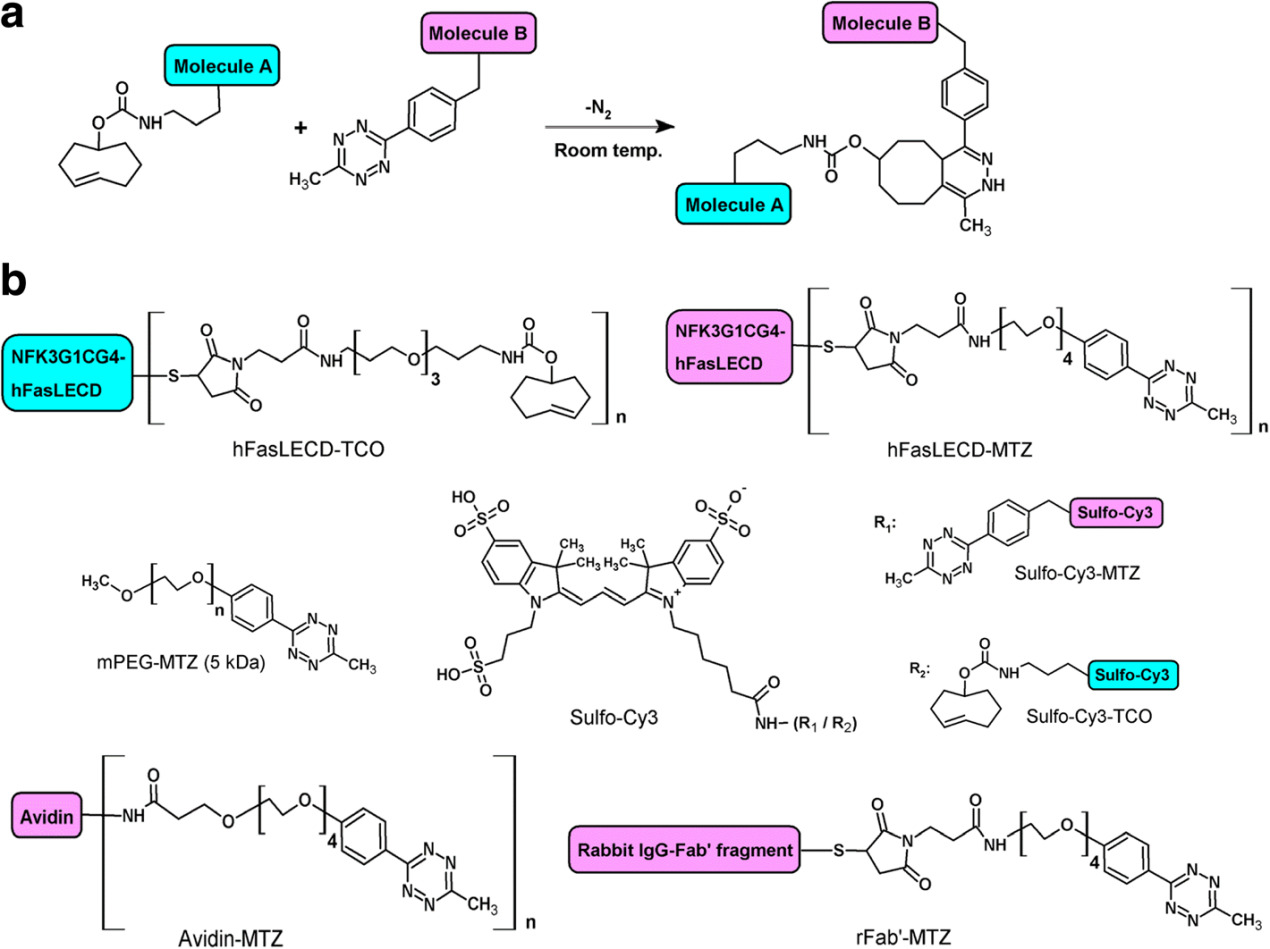
Schematic chemical structures of molecules relevant to the conjugation reactions between TCO- and MTZ-groups. **a** General conjugation reaction scheme. **b** Compounds used as the components in the TCO – MTZ conjugation reactions. With respect to the protein molecules, only TCO- and MTZ-group containing spacer arms are drawn as detailed chemical structures. The “n” after the square brackets indicates eit﻿her ﻿a ﻿﻿﻿repeat of units ﻿or the possible multiple conjugations

An inverse-electron-demand Diels-Alder reaction between TCO- and MTZ-groups, which proceeds at room temperature with the generation of nitrogen gas as the sole side product, was used for the conjugation reaction (Fig. 1a). In Fig. 1b, the schematic structures of the compounds used in the TCO – MTZ conjugation reactions in this study are summarized with each detailed chemical structure of the MTZ- or the TCO-group containing spacer arm. The conjugations were performed either between hFasLECD-TCO and an MTZ-group containing compound, or between hFasLECD-MTZ and a TCO-group containing compound. For the preparation of hFasLECD-TCO and hFasLECD-MTZ, the reactive cysteine residue in the N-terminal tag sequence of hFasLECD molecule was chemically modified with a large excess molar amount of *trans*-cyclooctene-PEG_3_-maleimide (TCO-PEG3-MAL) and methyltetrazine-PEG_4_-maleimide (MTZ-PEG4-MAL) reagents, respectively. In this study, NFK3G1CG4-hFasLECD, a revised hFasLECD derivative containing three additional lysine residues following the DYKDDDDK (FLAG) tag sequence as compared to NFG1CG4-hFasLECD [19] was exploited for the derivatization (Additional file 1a). NFK3G1CG4-hFasLECD was produced using a secretory expression system in *P. pastoris* as described in the previous papers [24, 25]. To date, the tertiary structure of a complex between hFasLECD and human decoy receptor 3 (DcR3) has been determined by X-ray crystallography, which serves as a model for hFasLECD – hFasRECD complex [26]. From a viewpoint of three-dimensional structure, the attachment site of the tag sequence was designed to locate not proximal to the receptor binding interface in order to avoid the interference with the specific recognition of hFasRECD (Additional file 1b). The additional lysine residues in the tag sequence were introduced to increase the isolelectric point value for making the isolation of the hFasLECD derivative from other impurities in the culture medium easier than the case of the original derivative at the initial purification step using a simple stepwise salt-gradient elution (Additional file 1c).

**Fig. 2:**
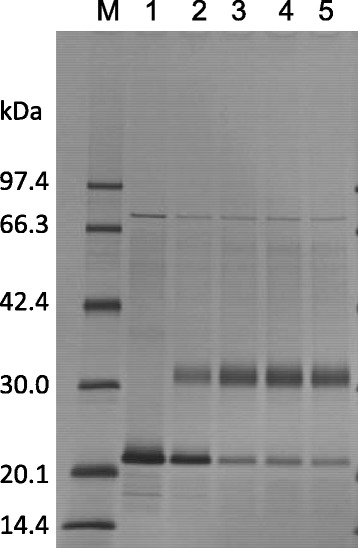
SDS-PAGE analysis of the conjugation reaction between hFasLECD-TCO and mPEG-MTZ. Lanes: M, molecular-weight size markers; 1, before reaction, 2–5, after reaction, (2: 0.5, 3: 1.0, 4: 1.1 and 5: 1.5 M excess amounts of mPEG-MTZ were reacted with hFasLECD-TCO)

As a preliminary evaluation of the conjugation efficiency using the TCO – MTZ reaction, the percentage of the reactive TCO-groups, introduced by the modification of NFK3G1CG4-hFasLECD with a large excess molar amount of TCO-PEG3-MAL, was evaluated by the reaction of hFasLECD-TCO with 0.5, 1.0, 1.1 and 1.5 M excess amounts of methyltetrazine conjugated mPEG(5 kDa) (mPEG-MTZ) (Fig. 1b). The ratio of the conjugated product to non-conjugated sample remained almost the same among the experiments using from 1.0 to 1.5 M excess amounts of mPEG-MTZ reagent (Fig. 2). This suggested that the use of 1.0–1.5 M excess amounts of mPEG-MTZ was enough to saturate the reaction efficiency. The maximum percentage of the conjugated product was estimated to be approximately 80% by a densitometry analysis of the protein bands on the SDS-PAGE gel.

### Preparation and characterization of sulfo-Cy3-TM-hFasLECD and sulfo-Cy3-MT-hFasLECD

**Fig. 3:**
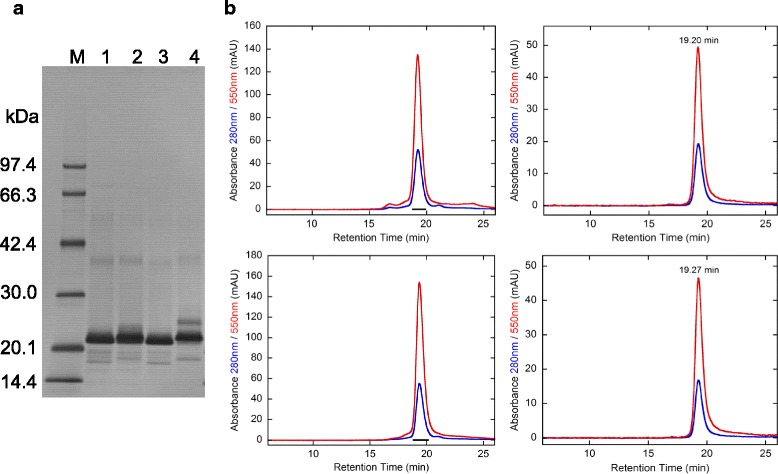
Conjugation of hFasLECD with sulfo-Cy3. **a** SDS-PAGE analysis of the conjugation reaction. Lanes: M, molecular-weight size markers; 1, hFasLECD-MTZ alone; 2, hFasLECD-MTZ reacted with a 1.3 M excess amount of sulfo-Cy3-TCO; 3, hFasLECD-TCO alone; 4, hFasLECD-TCO reacted with a 1.4 M excess amount of sulfo-Cy3-MTZ. **b** High-performance size-exclusion chromatography profiles. Upper panels, hFasLECD-TCO reacted with a 1.4 M excess amount of sulfo-Cy3-MTZ; lower panels, hFasLECD-MTZ reacted with a 1.3 M excess amount of sulfo-Cy3-TCO; left panels, crude samples; right panels, purified samples

**Fig. 4:**
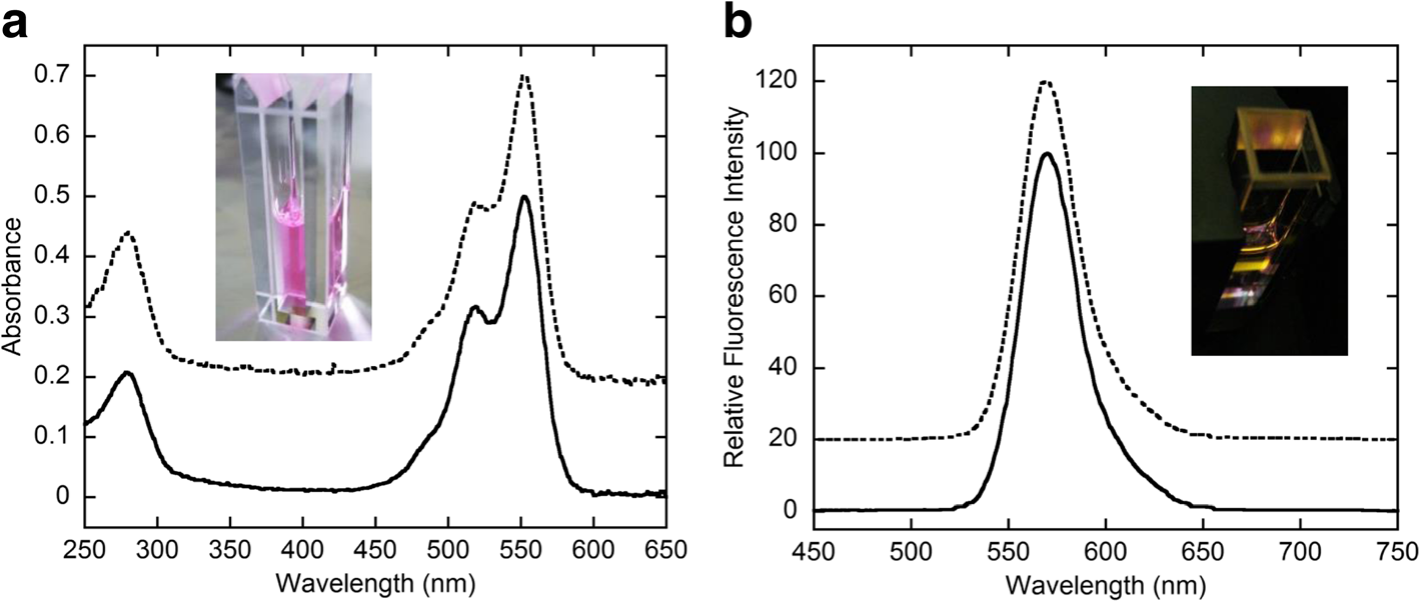
Spectroscopic analysis of sulfo-Cy3 conjugated hFasLECDs. Dot lines, Sulfo-Cy3-MT-hFasLECD; solid lines, Sulfo-Cy3-TM-hFasLECD. **a** UV-Vis spectra. The absorbance of Sulfo-Cy3-MT-hFasLECD was expressed as values of the experimental data plus 0.2. Insert, an appearance under white light. **b** Fluorescence emission spectra excited at 552 nm. The relative fluorescence intensity of Sulfo-Cy3-MT-hFasLECD was expressed as values of the experimental data plus 20. Insert, a fluorescence emission observed in the measurement cuvette

The conjugation reactions were conducted using 1.3–1.4 M excess amounts of the sulfo-Cy3 reagents, i.e. sulfo-Cy3-methyltetrazine (Sulfo-Cy3-MTZ) and sulfo-Cy3-*trans*-cyclooctene (Sulfo-Cy3-TCO) (Fig. 1b), relative to either hFasLECD-TCO or hFasLECD-MTZ. The reaction mixtures for generating the two alternative types of sulfo-Cy3-hFasLECD conjugates were analyzed by SDS-PAGE (Fig. 3a) and the high-performance size-exclusion chromatography (Fig. 3b, left panels). The SDS-PAGE analysis showed that the protein bands of both reaction mixtures consisted of a single major dense band at around 21–22 kDa and some other minor bands. The high-performance size-exclusion chromatography analysis of the reaction mixtures presented a single major peak showing the absorbance at both 280 nm and 550 nm, and the retention time of 19.2–19.3 min, in either case. These results indicated that the reaction products were nearly homogeneous. In Fig. 3b (right panels), the chromatography profiles about the final samples after purification are shown. The ratios of the peak absorbance at 550 nm to that at 280 nm concerning the purified samples after fractionation were 2.6 and 2.8 with regard to the sulfo-Cy3-MTZ conjugated hFasLECD-TCO (Sulfo-Cy3-MT-hFasLECD) and the sulfo-Cy3-TCO conjugated hFasLECD-MTZ (Sulfo-Cy3-TM-hFasLECD), respectively. This suggested that an effective conjugation of a sulfo-Cy3 moiety to the hFasLECD derivative was attained using even only 1.3–1.4 M excess amounts of the modification reagents in either case. In Fig. 4, ultraviolet-visible (UV-Vis) absorption spectra and fluorescence emission spectra of the purified conjugate samples are presented. Both samples showed the spectra with the maximum absorption peak at 551–552 nm and the fluorescence emission peak at 570 nm, which are the functional characteristics of sulfo-Cy3 group. The estimated conjugation number of the fluorochrome per a single hFasLECD trimer, calculated from the ratio of the absorbance at 280 nm to that at 552 nm, were 1.5 and 1.6 for Sulfo-Cy3-MT-hFasLECD and Sulfo-Cy3-TM-hFasLECD, respectively.

**Fig. 5:**
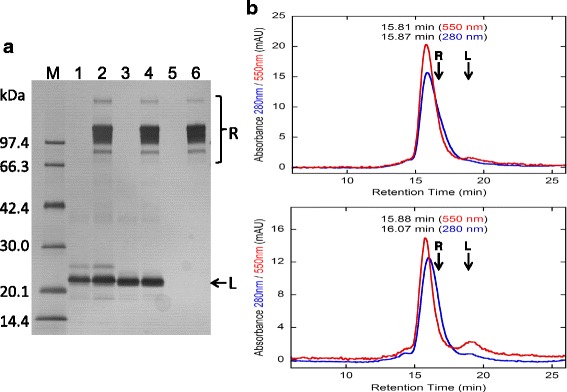
Complex formation of sulfo-Cy3 conjugated hFasLECDs with hFasRECD-Fc. L and R indicate the positions of sulfo-Cy3 conjugated hFasLECDs and hFasRECD-Fc, respectively. **a** SDS-PAGE analysis of the receptor mediated co-immunoprecipitation. Lanes: M, molecular-weight size markers; 1 and 2, sulfo-Cy3-MT-hFasLECD; 3 and 4, sulfo-Cy3-TM-hFasLECD; 5 and 6, buffer; 1 and 3, purified samples; 5, buffer alone sample; 2, 4 and 6, co-immunoprecipitated materials. **b** High-performance size-exclusion chromatography profiles. The mixtures of sulfo-Cy3 conjugated hFasLECDs (7.5 μg each) and hFasRECD-Fc (19.4 μg each) were analyzed. Upper panel, sulfo-Cy3-MT-hFasLECD; lower panel, sulfo-Cy3-TM-hFasLECD

The capability of the Sulfo-Cy3-hFasLECDs to form a specific complex with hFasRECD-Fc was examined using a receptor-mediated co-immunoprecipitation experiment and the high-performance size-exclusion chromatography analysis. Both conjugate samples were shown to retain a strong binding activity toward hFasRECD-Fc using the co-immunoprecipitation experiments (Fig. 5a). The major peaks showing the absorbance of 550 nm appeared at an earlier position than the retention time of hFasRECD-Fc showing 280 nm absorbance alone (Additional file 2), in both cases. This together with the non-existence of a large peak of the free ligand component in the size-exclusion chromatography profiles clearly showed the complex formation (Fig. 5b). However, a small difference in the chromatography profile was detected between Sulfo-Cy3-MT-hFasLECD (upper) and Sulfo-Cy3-TM-hFasLECD (lower). A slightly larger delay in the peak retention time of the absorbance at 280 nm from that at 550 nm, coincided with the existence of a higher peak at the elution position of the free ligand component, was observed for Sulfo-Cy3-TM-hFasLECD as compared with Sulfo-Cy3-MT-hFasLECD. This result suggested a stronger binding activity of Sulfo-Cy3-MT-hFasLECD than Sulfo-Cy3-TM-hFasLECD toward hFasRECD-Fc. Consequently, hFasLECD-TCO was selected as the component molecule in the following conjugation experiments with functional proteins.

### Isolation of avidin-hFasLECD and rFab’-hFasLECDs

**Fig. 6:**
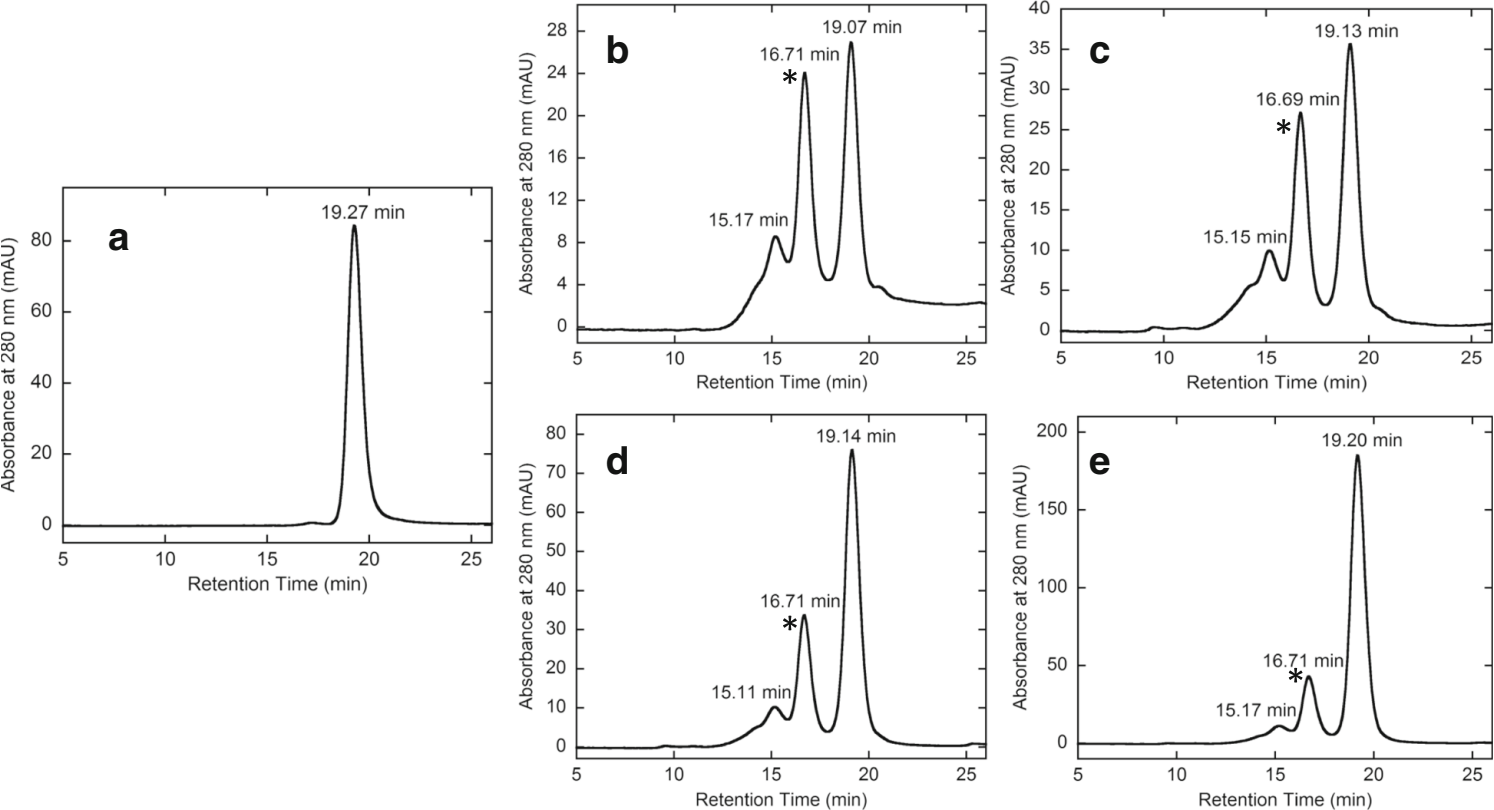
Conjugation reaction of Avidin-MTZ and hFasLECD-TCO analyzed by high-performance size-exclusion chromatography. Panels: **a**, Avidin-MTZ alone; **b**–**e**, reaction mixtures (b: 1.0, c: 1.2, d: 1.5 and e: 3.0 M excess amounts of Avidin-MTZ were reacted with hFasLECD-TCO). Retention time of each peak is shown

**Fig. 7:**
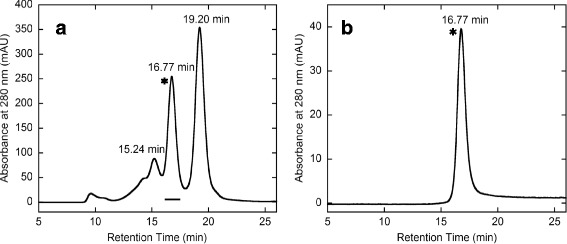
Isolation of Avidin-hFasLECD conjugate by high-performance size-exclusion chromatography. Panels: **a**, reaction mixture after quenching with TCO-Amine. The peak fraction shown in under bar was collected; **b**, isolated sample

A high-performance size-exclusion chromatography was used for the evaluation of the progress of the conjugation reaction between hFasLECD-TCO and the MTZ-group(s) containing derivatives of proteins. The Avidin-MTZ sample showing a single peak in the high-performance size-exclusion chromatography (Fig. 6, panel a) was used for the conjugation with hFasLECD-TCO. It was reasonable to consider that the Avidin-MTZ molecule possessed multiple MTZ groups (Fig. 1b), since the sample was synthesized by the reaction of native avidin, existing as a homotetramer containing nine lysine residues per monomer unit, with eightfold molar excess amount of methyltetrazine-PEG_4_-sulfo-N-hydroxysuccinimide ester (MTZ-PEG4-sNHS). As a trial conjugation experiment, a series (1.0, 1.2, 1.5 and 3.0 M excess amounts) of Avidin-MTZ were reacted with hFasLECD-TCO to examine the effect of molar ratio on the product profile in the reaction mixture. In Fig. 6 (panels b – e), the profile of each reaction mixture in the high-performance size-exclusion chromatography is shown. The essential pattern of the chromatography profiles among them resembled to each other. Of note, a distinct peak (marked with an asterisk in the Figure panels) with the retention time of 16.69–16.71 min was always appeared. Judging from the retention time, this peak was thought to contain the one to one conjugate between avidin-MTZ and hFasLECD-TCO. The corresponding peak fraction sample was isolated as a single peak from the reaction mixture after quenching with an excess amount of *trans*-cyclooctene-amine hydrochloride salt (TCO-Amine) (Fig. 7).

**Fig. 8:**
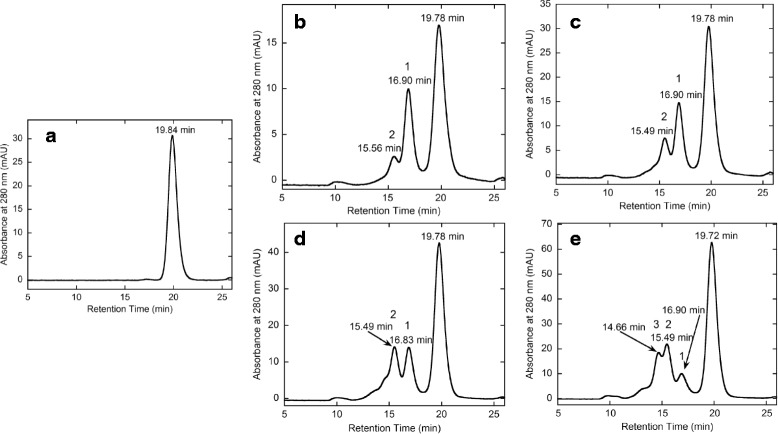
Conjugation reaction of rFab’-MTZ and hFasLECD-TCO analyzed by high-performance size-exclusion chromatography. Panels: **a**, rFab-MTZ alone; **b**–**e**, reaction mixtures (**b**, 1.0; **c**, 2.0; **d**, 3.0 and **e** 5.0 M excess amounts of rFab’-MTZ were reacted with hFasLECD-TCO). Retention time of each peak is shown

**Fig. 9:**
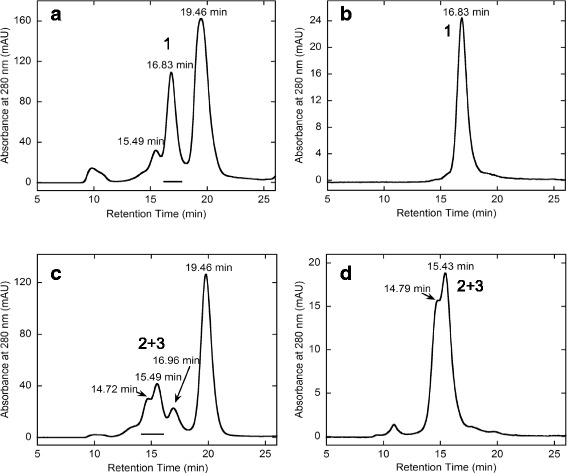
Isolation of rFab’-hFasLECD conjugates by high-performance size-exclusion chromatography. Panels: **a** and **c**, reaction mixture after quenching with MTZ-PEG4-Amine. (**a**, 1.0 M excess amount of rFab’-MTZ; **c**, 5.0 M excess amount of rFab’-MTZ); **b**, isolated sample from **a**; **d**, isolated sample from **c**. Retention time of each peak is shown

On the other hand, the rFab’-MTZ molecule was considered to possess a single MTZ group (Fig. 1b), since it was synthesized by the modification of the terminal single cysteine residue with a large excess molar amount of MTZ-PEG4-MAL. A series (1.0, 2.0, 3.0 and 5.0 M excess amounts) of the purified rFab’-MTZ sample showing a single peak in the high-performance size-exclusion chromatography analysis (Fig. 8, panel a) were used for the trial conjugation reactions with hFasLECD-TCO to examine the effect of the molar ratio on the product profile (Fig. 8, panels b – e). The chromatography profile significantly depended on the molar ratio of rFab’-MTZ relative to hFasLECD-TCO. Three distinct peaks (designated as peaks 1, 2 and 3 according to the numbering in the Fig. 8, panels b - e) gradually emerged as the molar excess amount value of rFab’-MTZ increased. As judged by the decrease in the retention time showing the increase in the molecular weight in the order of peak 3 > peak 2 > peak 1, the emergence of these peaks was considered to correspond to the single, double and triple conjugation of rFab’-MTZ per a single hFasLECD-TCO timer molecule, respectively. Among them, a single peak fraction consisting of peak 1 and a combined fraction mainly composed of the peaks 2 and 3 were isolated after quenching with an excess amount of methyltetrazine-PEG4-amine hydrochloride salt (MTZ-PEG4-Amine) (Fig. 9).

### Characterization of the isolated samples of avidin-hFasLECD and rFab’-hFasLECDs

**Fig. 10:**
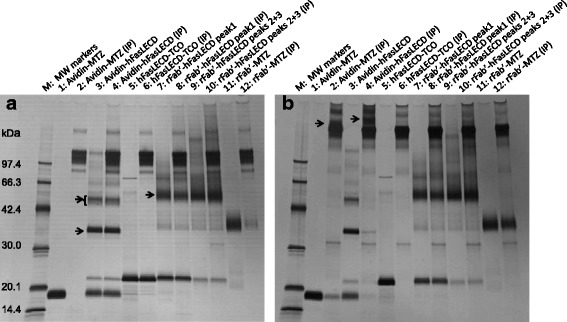
SDS-PAGE analysis of co-immunoprecipitation experiments under non-reducing condition. Panels: **a**, receptor-mediated co-immunoprecipitation using hFasRECD-Fc; **b**, antibody-mediated co-immunoprecipitation using biotin conjugated goat anti-rabbit IgG H&L. Lanes (in both panels): M, molecular-weight size markers; 1 and 2, Avidin-MTZ alone; 3 and 4, Avidin-hFasLECD conjugate; 5 and 6, hFasLECD-TCO alone; 7 and 8, rFab’-hFasLECD conjugate (the peak 1 fraction); 9 and 10, rFab’-hFasLECD conjugate (the combined fractions of peaks 2 and 3); 11 and 12, rFab’-MTZ alone; 1, 3, 5, 7, 9 and 11, examined samples; 2, 4, 6, 8, 10 and 12, precipitated materials (IP)

In Fig. 10, panels a and b present the results of receptor-mediated and antibody-mediated co-immunoprecipitation experiments using hFasRECD-Fc and biotin-conjugated goat anti-rabbit IgG H&L as the specific binding linker between the examined molecules and Protein G-conjugated magnetic beads, respectively. Each pair of the examined samples and the corresponding precipitated materials was arranged in parallel and analyzed using a non-reducing SDS-PAGE. Avidin-MTZ (lane 1), hFasLECD-TCO (lane 5) and rFab’-MTZ (lane 11) migrated at the positions of approximately 17 kDa, 21 kDa and 40 kDa, respectively. The isolated sample of avidin-hFasLECD conjugate was resolved into several discrete bands (lane 3).

The multiple bands were considered to be arising from the fact that non-denatured avidin-MTZ and non-denatured hFasLECD-TCO existed as a homotetramer and a homotrimer, respectively. Both of them should be dissociated into identical subunits under the denaturing SDS-PAGE condition. Judged by the molecular weights, the densest band at the position between the molecular-weight markers of 30.0 kDa and 42.4 kDa (the lower arrow in lane 3 of panel a) was considered to be the major conjugation product consisted of one avidin subunit and one hFasLECD subunit. The broad, weaker band migrated between 42.4 kDa and 66.3 kDa (the upper arrow in lane 3 of panel a) was thought to be the conjugation product consisted of one avidin subunit and two hFasLECD subunits, in which some conformational variations to affect the migration position of the band can exist depending on the attachment sites on the avidin subunits. On the other hand, rFab’-MTZ existed as a monomer protein, and therefore the broad, major band (the arrow in lane 7 of panel a) migrated between the positions of molecular-weight markers of 42.4 kDa and 66.3 kDa was considered to be the one to one conjugation product between the rFab’ domain and the hFasLECD subunit (lanes 7 and 9 in both panels).

In the co-immunoprecipitation experiment using hFasRECD-Fc as the specific binder (Fig. 10, panel a), all the conjugated samples and hFasLECD-TCO alone sample were precipitated (lanes 4, 6, 8 and 10), indicating the specific binding of the hFasLECD components to hFasRECD-Fc. This showed the functional integrity of the hFasLECD components in the conjugated samples. Avidin-MTZ alone sample did not react at all as expected (lane 2). A weak signal was also observed for Fab’-MTZ alone sample (lane 12), which should be ascribed to the specific, but weak direct interaction between rabbit Fab’ domain and Protein G [27]. On the other hand, in the experiment using biotin conjugated goat anti-rabbit IgG H&L as the specific binder (Fig. 10, panel b), rFab’ conjugated hFasLECD samples and rFab’-MTZ alone sample showed strong signals (lanes 8, 10 and 12), indicating the specific binding of the rFab’ domains to the antibody. This presented the structural integrity of Fab’ domains in the conjugates, since the antibody was isolated by affinity chromatography using the antigen coupled to agarose beads, and then conjugated to biotin [28]. The precipitation of Avidin-MTZ alone sample (lane 2) and avidin-hFasLECD conjugate sample (lane 4) was also considered to occur. However, most of the precipitated product migrated at a higher position than the antibody (the arrows in lanes 2 and 4 of panel b), and only weak bands were observed at each original positions of the major bands of the examined samples. This retarded migration can be explained by the formation of a large molecular-weight complex between the dissociated monomeric avidin subunits and the multiple biotin moieties pre-conjugated to the antibody. This type of complex was also reported to be fairly stable under the non-reducing SDS-PAGE condition in a capillary gel electrophoresis [29]. As expected, hFasLECD-TCO alone sample did not precipitate at all (lane 6).

**Fig. 11:**
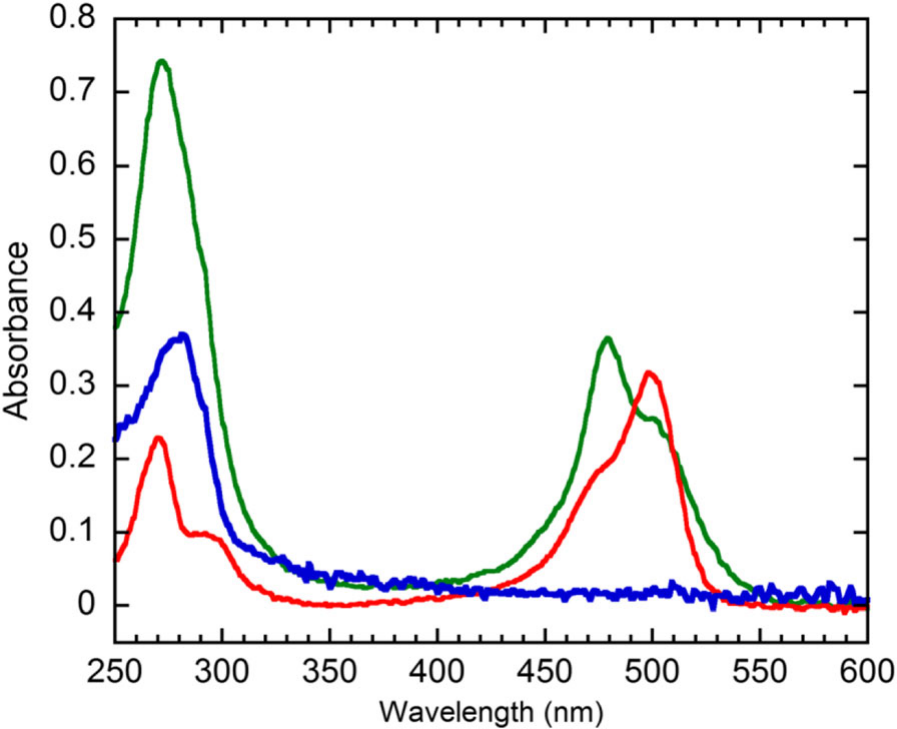
Detection of complex formation between Avidin-hFasLECD conjugate and ATTO495-Biotin. Lines: green, isolated complex between Avidin-hFasLECD conjugate and ATTO495-Biotin; blue, Avidin-hFasLECD conjugate alone; red, ATTO495-Biotin alone

The biotin moiety binding of the isolated avidin-hFasLECD sample under the non-denatured condition was examined by mixing it with ATTO495-Biotin in a buffer solution of pH 7.5 without any detergent. As shown in Fig. 11, both an emergence of significant absorption peak with the maximum at 479 nm (green line) not detected for the conjugated protein sample alone (blue line) and a remarkable increase in the absorbance peak at 272 nm as compared to the conjugated sample alone (blue line) were observed for the complex sample (green line). The result evidently showed the existence of biotin binding activity of the isolated avidin-hFasLECD conjugate sample in a native state under the physiological pH buffer condition. In this binding event, a significant shift of the maximum absorption wavelength of the complex (479 nm, green line) from that of the free ATTO495-Biotin (498 nm, red line), concomitant with a remarkable quenching of fluorescence to approximately 10% of the original free ligand, was observed. A similar quenching phenomenon was also reported in the specific binding of biotin-fluorescein towards avidin in neutral pH buffer solution [30].

## Discussion

The reaction between TCO- and MTZ-groups can proceed efficiently in buffers of physiological pH using a small excess molar amount of the reagents relative to the molecule to be modified. Therefore, it provides a good opportunity of site-specific chemical conjugations for attaching valuable molecules, such as expensive low molecular-weight compounds or a variety of biomolecules including precious functional proteins [21]. In this study, we investigated the possibilities of the site-specific conjugation of an hFasLECD derivative with sulfo-Cy3 fluorochrome, avidin and rabbit Fab’-domain as the model experiments.

Two alternative types of sulfo-Cy3 conjugated hFasLECD were obtained using 1.3–1.4 excess molar amounts of either MTZ-group or TCO-group containing derivatives of sulfo-Cy3. These sulfo-Cy3 group containing reagents are fairly stable in physiological pH buffer conditions, and are commercially available at the comparable prices to the corresponding maleimide derivatives. In general, the maleimide derivatives of fluorochromes, such as fluorescein-5-maleimide, are recommended to use a large molar excess amount, typically 25-fold molar excess amount, for the efficient conjugation with free thiol groups in cysteine residues of the target proteins [31]. The obtained conjugation products in the present study showed sound fluorescence emission spectra derived from the attached sulfo-Cy3 groups together with the retention of the strong hFasRECD-Fc binding activity. This proved the possibility of less expensive derivatization of hFasLECD with valuable fluorochromes, although some decrease in the conjugation number resulted from the two step conjugation procedures, composed of the first introduction of either TCO- or MTZ-group into the hFasLECD derivative and the following conjugation of the fluorochrome using the TCO – MTZ reaction, should be taken into account.

In regard to the modification of hFasLECD-TCO with protein molecules, i.e. avidin-MTZ and rFab’-MTZ, significant amounts of the conjugation products were produced in the reaction mixtures using 1.0 to 3.0 M excess amounts of avidin-MTZ and 1.0 to 5.0 M excess amounts of rFab’-MTZ. The isolated samples showed a strong hFasRECD-Fc binding activity as well as the functional and the structural integrities of the other component in the conjugation products. These results revealed that the conjugation of both avidin and rFab’ domain to hFasLECD in parallel with maintaining the original functions of both protein components was possible using relatively small excess molar excess amounts of the derivative of each protein, via the reaction between TCO – MTZ groups. However, considerably larger amounts of the remaining non-conjugated molecules were observed in the reaction mixtures consisted of nearly equivalent molar amounts of the TCO- and the MTZ- components as compared to the case of mPEG-MTZ. The results indicated that the conjugation reactions of hFasLECD-TCO with the MTZ derivatives of the proteins did not always proceed quantitatively, which required an efficient isolation step of the conjugated products for further characterization. This suggested that a steric hindrance derived from the bulky three-dimensional structures of the proteins became a substantial restriction factor for the efficiency of the TCO-MTZ reactions in the actual protein-protein conjugations.

The site-specific sulfo-Cy3 conjugates of hFasLECD may be useful for the evaluation of the cell-surface density of hFasR including that expressed on cancer cells, which might reflect the therapeutic response to clinical cytotoxic drugs [11–14]. The conjugation methodology toward the hFasLECD derivative using the TCO – MTZ reaction should also be applicable to other useful fluorochromes for the quantification of cell-surface receptors by flow cytometry. The receptor binding event is essentially based on the direct hFasRECD recognition function of hFasLECD in native states. Thus, the applications are considered to be suitable for the fluorescent detection of cell-surface hFasR in viable cells, which can eliminate the background and the false positive reactions derived from the modifications of cell surface caused by fixation [32, 33]. Concerning the modifications with functional proteins, the possible conjugation with Fab’ fragments and other related domains of monoclonal antibodies specific to surface antigens will provide an opportunity for the targeting of hFasLECD to diseased cells. The avidin-hFasLECD conjugate holds the potential to bind any biotin conjugated molecules, such as biotinylated monoclonal antibodies targeted to malignant cells. The avidin-hFasLECD conjugate may be also applicable to enzyme-linked immunosorbent assays in cooperation with biotin conjugated enzymes. Taken together, the above application possibilities of site-specific chemical conjugates of hFasLECD as novel molecular tools will lead to the development of the improved diagnostic systems and the effective treatment methods toward serious disorders, in which the cell-surface hFasR plays critical roles [1, 34, 35].

## Conclusions

In this study, we devised a new fundamental procedure for the preparation of site-specific chemical conjugates of hFasLECD with a valuable low molecular-weight compounds and precious functional proteins using the TCO – MTZ conjugation reaction. The chemical reaction required much less molar excess amount of the molecules to be conjugated as compared to the conventional thiol – maleimide reaction used in the previous study [20]. The isolated samples maintained the functional and the structural integrities of both components in the conjugates, which will lead to the development of novel molecular tools with potentials for various medical applications.

## Methods

### Materials

A gene of hFasLECD (amino acid residues, 139–281) containing double substitution mutations (N184Q and N250Q) with an N-terminal FLAG-(LysLysLysGlyCysGlyGlyGlyGly) tag sequence (NFK3G1CG4-hFasLECD) was constructed by introducing nine nucleotide bases (AAGAAGAAG) insertion mutation into the gene of NFG1CG4-hFasLECD. The production of NFK3G1CG4-hFasLECD in a *P. pastoris* secretory expression system was conducted as described previously [19]. hFasRECD-Fc was produced in a *baculovirus* – *Bombyx mori* expression system and purified as described in the previous paper [36]. Avidin from egg white (for biochemistry), normal rabbit IgG whole molecule (purified by Protein A), Pepsin from porcine stomach, 2-aminoethanethiol hydrochloride salt and washing buffer reagents used in the immunoprecipitation experiments were obtained from Wako Pure Chemicals, Ind. Biotin conjugated goat anti-rabbit IgG H&L (ab207995) and ATTO495-Biotin were from Abcam Co. and ATTO-TEC GmbH, respectively.

TCO-PEG3-MAL, MTZ-PEG4-MAL, mPEG-MTZ, MTZ-PEG4-sNHS, Sulfo-Cy3-MTZ, TCO-Amine and MTZ-PEG4-Amine were purchased from Click Chemistry Tools. Sulfo-Cy3-TCO was from AAT Bioquest, Inc. A product of Protein G conjugated magnetic beads (SureBeads Protein G) was obtained from Bio-Rad Laboratories. A high-performance size-exclusion chromatography column (Superdex 200 Increase 10/300 GL, bed dimensions: 10 × 300 mm, bed volume: approximately 24 ml) was purchased from GE healthcare. Other chemical reagents and devices of biochemical grade were as described in the previous paper [20]. Chemical structures were drawn using ACD/Chemsketch (Free ware) 2016.1.1. A densitometry analysis of the protein bands on an SDS-PAGE gel was performed using Image J [37].

In the following experiments, all protein sample concentration was conducted using an Amicon Ultra 15 [molecular-weight cut off (MWCO): 10 kDa] device by the centrifugation of 5000 G at 277 K. The size-exclusion chromatography fractionation by a disposable column in gravity-flow mode was performed using a PD-10 column (GE healthcare). High-performance size-exclusion chromatography was carried out using a Superdex 200 Increase 10/300 GL column under the conditions of 50 mM tris-hydrochloride containing 150 mM sodium chloride (pH 7.5) [50 mM Tris-HCl plus 150 mM NaCl (pH 7.5)] as the elution buffer and flow rate of 0.75 ml / min. In these conditions, the peak retention time of Ovalbumin (43 kDa), Aldolase (158 kDa) and Thyrogloblin (669 kDa) was 20.10 min, 17.73 min and 12.61 min, respectively. All sample solutions of the TCO- and MTZ-groups containing compounds were kept frozen at 253 K until use. Protein concentration was determined by a BCA protein assay kit using bovine serum albumin as the standard sample. SDS-PAGE analyses were performed using a 10–20% gradient gel and the protein bands were visualized by silver stain.

### Preparation of TCO- and MTZ-derivatives of NFK3G1CG4-hFasLECD

The NFK3G1CG4-hFasLECD sample used for the preparation of either TCO- or MTZ-derivative was purified by a cation-exchange column chromatography (Hi-Trap SP, 5 ml) as described [19]. The protein concentration of the purified sample was determined to be 9.1 mg / ml. Freshly prepared twenty-fold molar excess amount each of TCO-PEG3-MAL and MTZ-PEG4-MAL in dehydrated dimethyl sulfoxide (dry DMSO) was used for the modification reactions to obtain the TCO- and MTZ-derivatives, respectively. Other details in experimental procedures were the same as described for the preparation of fluorescein 5-maleimide derivative in the previous paper [20], except for the substitution of the final purification step using the high-performance size-exclusion chromatography with the concentration step after the second size-exclusion chromatography in a gravity-flow mode. Typically, final recovery yields of the purified samples were 5.9 mg and 6.9 mg with respect to the TCO-derivative (hFasLECD-TCO) and the MTZ-derivatives (hFasLECD-MTZ) starting from 12 mg each of the purified NFK3G1CG4-hFasLECD samples, respectively.

### Reactions of hFasLECD-TCO with mPEG-MTZ

Twenty μl (50 μg, 2.8 nmoles as the monomer subunit) each of hFasLECD-TCO (2.5 mg/ml) in 50 mM sodium acetate (pH 5.5) was mixed with 1.4 μl (0.5 M excess), 2.8 μl (1.0 M excess), 3.1 μl (1.1 M excess) or 4.1 μl (1.5, molar excess) of mPEG-MTZ (5 kDa) solution (5 mg / ml in deionized water). The reaction mixture was incubated for 1 h at 297 K, and then subjected to an SDS-PAGE analysis.

#### Preparation of sulfo-Cy3 conjugated NFK3G1CG4-hFasLECDs

For the conjugation with sulfo-Cy3-MTZ, 3.3 ml (5.5 mg, 0.30 μmole as the monomer subunit) of hFasLECD-TCO solution in 50 mM sodium acetate (pH 5.5) was mixed with 330 μl (0.41 μmole, a 1.4 M excess amount) of sulfo-Cy3-MTZ solution (1.1 μmoles / ml in deionized water). The reaction mixture was incubated for 1 h at 297 K. The same procedure was conducted using 3.3 ml (6.5 mg, 0.36 μmole as the monomer subunit) of hFasLECD-MTZ solution in 50 mM sodium acetate (pH 5.5) and 436 μl (0.48 μmole, a 1.3 M excess amount) of sulfo-Cy3-TCO solution (1.1 μmoles / ml in deionized water) for the conjugation of sulfo-Cy3-TCO. In either case, the reaction mixture after the incubation period was immediately resolved by two tandem steps of the chromatography in a gravity flow mode using 50 mM sodium acetate (pH 5.5) as the elution buffer. In the first resolving step, the reaction mixture sample was divided into two equivalent volume aliquots for a single application to the column, and one ml each fraction was collected into the reservoirs. The combined early four fractions eluted as pink, clear solutions were concentrated to approximately 2.0 ml. Then, the concentrate was subjected to the second resolving step for removing the remaining low molecular-weight contaminants completely. Finally, the sample was purified by the high performance size-exclusion chromatography. A 230 μl each aliquot of the sample was applied to the column in an individual run. The single main peak fractions showing the absorbance of 280 nm and 550 nm at the identical retention time were collected. The fractionated samples were combined and concentrated to a fluorescent deep pink, clear solution. The final recovery yield and the protein concentration in parenthesis were 2.8 mg (1.3 mg / ml) and 3.1 mg (0.92 mg / ml) with regard to Sulfo-Cy3-MT-hFasLECD and Sulfo-Cy3-TM-hFasLECD, respectively. The samples were kept frozen at 253 K in the dark until use, and then subjected to the SDS-PAGE analyses, the spectroscopic measurements and the experiments for the detection of complex formation with hFasRECD-Fc using the co-immunoprecipitation and the high-performance size-exclusion chromatography analyses.

### Preparation of avidin-hFasLECD

Avidin-hFasLECD was synthesized by the conjugation of Avidin-MTZ with hFasLECD-TCO. Avidin-MTZ (Fig. 1b) was prepared by the reaction of a commercially available biochemical grade avidin from chicken egg-white with eightfold molar excess amount of MTZ-PEG4-sNHS as follows. Ten mg of avidin was dissolved in 2.0 ml of 0.1 M sodium hydrogen carbonate (pH 8.3), then 75 μl of MTZ-PEG4-sNHS solution (2 mg in 200 μl deionized water) prepared immediately before the reaction was added. The reaction mixture was incubated for 4 h at 301 K. After that, the reaction mixture was quenched with 140 μl of 1 M Tris HCl (pH 7.5) and further incubated for 15 min. The quenched sample was resolved by the size-exclusion chromatography in a gravity-flow mode using 50 mM Tris-HCl plus 150 mM NaCl (pH 7.5) as the elution buffer. The same resolution step was repeated again to remove the low molecular-weight contaminants containing MTZ group completely. The recovered sample was concentrated to 2.4 ml (4.3 mg/ml) of a pale pink, clear solution, and used as the sample for the following conjugation reactions.

Initial attempts of the conjugation reaction between Avidin-MTZ and hFasLECD-TCO were performed by mixing 10 μl, 20 μl or 30 μl of hFasLECD-TCO solution [2.52 mg/ml in 50 mM sodium acetate (pH 5.5)] with a series (1.0, 1.2, 1.5 or 3.0 M excess amount) of Avidin-MTZ solutions [4.3 mg/ml in 50 mM Tris-HCl plus 150 mM NaCl (pH 7.5)], and incubated for 1 h at 301 K. Each reaction mixture was diluted to 200 μl with 50 mM Tris-HCl plus 150 mM NaCl (pH 7.5) buffer¸ and then subjected to an analysis using the high performance size-exclusion chromatography. A large scale conjugation reaction under the condition of 1.5 fold excess molar amount of Avidin-MTZ relative to hFasLECD-TCO was conducted by mixing 1.1 ml (2.7 mg, 70 nmoles) of Avidin-MTZ solution with 1.0 ml (2.5 mg, 46 nmoles) of hFasLECD-TCO solution. The reaction mixture was incubated for 1 h at 299 K, and then quenched with 23 μl of 30 mM TCO-Amine solution (3.9 mg in 0.5 ml of deionized water) by incubating for further 1 h. The final colorless, clear reaction mixture after the quenching reaction was applied to a single step of the size-exclusion chromatography in a gravity-flow mode to remove the low molecular-weight contaminants, and then 230 μl aliquots of the recovered sample were resolved by the high performance size-exclusion column chromatography to obtain single peak fractions. All isolated fractions were combined together and concentrated to 1.4 ml for the analyses in the following experiments (recovery yield, 1.5 mg).

### Preparation of rFab’-hFasLECDs

rFab’-hFasLECDs were synthesized by the conjugation of rFab’-MTZ with hFasLECD-TCO. The rFab’ domain was obtained essentially according to the procedures described in the previous papers [38, 39]. Thirty five mg of the commercially available Protein A purified normal rabbit IgG whole molecule in 3.5 ml of 0.1 M sodium acetate containing 0.1 M sodium chloride buffer (pH 4.5) was digested with 1.6 mg of Pepsin from porcine stomach by incubating for 20 h at 310 K (Additional file 3a). The sample after the digestion was subjected to exchange the buffer with 50 mM Tris-HCl plus 150 mM NaCl (pH 7.5) by the size-exclusion column chromatography in a gravity-flow mode. Then, 230 μl aliquots of the sample were further fractionated by the high performance size-exclusion chromatography using the same buffer (Additional file 3b, left panel). The main peak fractions containing rF(ab’)_2_ were collected and combined to total sample volume of 32.0 ml. The sample was concentrated to 3.6 ml (5.4 mg/ml). To a half volume of this sample solution containing 9.8 mg (0.21 μmole) of rF(ab’)_2_, 48 μl of 0.5 M ethylenediaminetetraacetic acid sodium salt (EDTA-Na) (pH 8.0) and 240 μl of freshly prepared 100 mM 2-aminoethantiol hydrochloride solution in 50 mM Tris-HCl containing 10 mM EDTA-Na (pH 7.5) were added and incubated for 30 min at 310 K, for the conversion of rF(ab’)_2_ to rFab’. Then, the reaction mixture was immediately subjected to a size-exclusion chromatography column pre-equilibrated with 25 mM sodium phosphate containing 0.1 M sodium chloride and 5 mM EDTA-Na (pH 6.4) for buffer-exchange. The sample containing rFab’ was diluted to 9.7 ml with the same buffer, and freshly prepared MTZ-PEG4-MAL solution [10 mg (19 μmoles) in 0.97 ml of dry DMSO] was added. The reaction mixture was incubated for 3 h at 297 K, and then quenched with 22 μl of 1 M L-cysteine hydrochloride solution in deionized water by incubating further 1 h. The quenched reaction mixture was concentrated to 2.0 ml, and further subjected to the two tandem size-exclusion chromatography in a gravity-flow mode to remove the MTZ-group containing low molecular-weight contaminants completely. After that, the high-performance size-exclusion chromatography resolutions of 230 μl aliquots were performed to obtain the main peak fractions of rFab’-MTZ sample (Additional file 3b, right panel). The collected samples were combined and concentrated to 3.0 ml of pale pink, clear solution (recovery yield 6.9 mg, 2.3 mg/ml).

Initial attempts of the conjugation reaction between rFab’-MTZ and hFasLECD-TCO were performed by mixing 10 μl each of hFasLECD-TCO solution [2.5 mg / ml in 50 mM sodium acetate (pH 5.5)] with a series (1.0, 2.0, 3.0 or 5.0 M excess amount) of rFab’-MTZ solutions [2.3 mg / ml in 50 mM Tris-HCl plus 150 mM NaCl (pH 7.5)] and incubated for 1 h at 298 K. Each reaction mixture was diluted to 200 μl with 50 mM Tris-HCl plus 150 mM NaCl (pH 7.5) buffer for subjecting to an analysis by the high-performance size-exclusion column chromatography. Large scale conjugation reactions under the condition of 1.0 M excess and 5.0 M excess amounts of rFab’-MTZ relative to hFasLECD were conducted by mixing 1.2 ml (2.7 mg, 58 nmoles) of rFab’-MTZ solution with 1.3 ml (3.2 mg, 60 nmoles) of hFasLECD-TCO solution, and 1.5 ml (3.4 mg, 72 nmoles) of rFab’-MTZ solution with 0.31 ml (0.78 mg, 14 nmoles) of hFasLECD-TCO solution, respectively. Both reaction mixtures were incubated for 1 h at 298 K, and then quenched by incubating for further 1 h with 19 μl (in the 1.0 M excess amount reaction) and 4.8 μl (in the 5.0 M excess amount reaction) of 30 mM MTZ-PEG4-Amine solutions (5.0 mg in 0.42 ml of deionized water), respectively. The final pale pink, clear solutions were subjected to the size-exclusion chromatography in a gravity mode. Then, 230 μl aliquots were resolved using the high-performance size-exclusion column chromatography to obtain the fractionated samples. The isolated sample fractions combined together were concentrated to 1.0 ml (0.57 mg) and 0.88 ml (0.13 mg) with regard to the reaction using 1.0 M excess amount of rFab’-MTZ and that using the 5.0 M excess amount of rFab’-MTZ, respectively.

### Preparation of the complex between avidin-hFasLECD and ATTO495-biotin

1.2 ml (1.2 mg) of the isolated avidin-hFasLECD conjugate was mixed with 40 μl of ATTO495-Biotin solution (1 mg in 100 μl of Dry DMSO) and incubated for 2 h on ice. The mixture was resolved by the two tandem steps of chromatography in a gravity-flow mode in order to completely remove the free ATTO495-Biotin. The sample recovered in the second resolving step (0.84 mg, 240 μg / ml) was subjected to the experiment for detection of the complex.

### Spectroscopic measurements and estimation of conjugation number of sulfo-Cy3

UV-Vis absorption spectra in the range from 250 nm to 650 nm, a couple of independent measurements of absorption values at 280 nm and 552 nm used for the calculation of an estimated conjugation number of sulfo-Cy3 groups to hFasLECD and fluorescent spectra measurement under the condition of the excitation wavelength at 552 nm were performed as described in the previous paper [20]. All measurements were conducted under the sample concentrations of 125 μg / ml. In the calculation of the estimated conjugation number, the correction factor of sulfo-Cy3 group at 280 nm was set to 0.05, and the molar extinction coefficient of sulfo-Cy3 group was assumed as 150,000 [40]. The molar extinction coefficient of NFK3G1CG4-hFasLECD was obtained as 29,005 using the Prot Param tool on the EXPAsy Server [41].

### Detection of the complex formation

Detection of the specific binding activity of the isolated conjugates, i.e. sulfo-Cy3-hFasLECDs, Avidin-hFasLECD and rFab’-hFasLECDs, and the components of the conjugates, i.e. hFasLECD-TCO, Avidin-MTZ and rFab’-MTZ, (5.5 μg each) toward either the hFasRECD-Fc sample (8.8 μg) or biotin conjugated goat anti-rabbit IgG H&L (14.0 μg) were conducted using a Protein G conjugated magnetic beads (1.0 mg) as the precipitating agent by the receptor- or the antibody-mediated co-immunoprecipitation in 1.0 ml of 50 mM Tris-HCl plus 150 mM NaCl buffer (pH 7.5) containing 1% Nonidet P40 and 0.5% sodium deoxycholate, as described in the previous paper [25]. Another experiment for the detection of the complex formation between sulfo-Cy3-hFasLECDs and hFasRECD-Fc was also performed by the high-performance size-exclusion chromatography using the mixture solutions composed of sulfo-Cy3-hFasLECDs (7.5 μg each) and hFasRECD-Fc (19.4 μg) in 230 μl solution as described in the previous paper [20]. The UV-Vis spectra of the isolated complex sample of Avidin-hFasLECD conjugate with ATTO495-Biotin and the Avidin-hFasLECD conjugate alone sample were compared at the concentration of 240 μg / ml in 50 mM Tris-HCl plus 150 mM NaCl (pH 7.5). A solution of free ATTO495-Biotin showing the absorbance value at 495 nm (0.29) similar to that of the isolated complex sample (0.26) was also subjected to measurement for comparison of the absorption peak profiles.

## Additional files

Additional file 1: Preparation of NFK3G1CG4-hFasLECD. a) Gene structure of expression unit and detailed tag sequences. In the tag sequences, the introduced three lysine residues and the reactive cysteine residue used for chemical modification with either TCO-PEG3-MAL or MTZ-PEG4-MAL are shown in blue and red, respectively. AOX-1 P, *P. pastoris* alcohol oxidase 1 promoter region; α-Prepro, *Saccharomyces cerevisiae* α-factor secretion-signal sequence; Tag, tag sequence; hFasLECD (139–281, N184Q, N250Q), human Fas ligand extracellular domain containing deletion mutation from residue 103 to 138 and double substitution mutation (N184Q and N250Q); AOX-1 TT, *P. pastoris* alcohol oxidase 1 transcription termination region. b) Three dimensional structure of hFasLECD-hDcR3 complex [26]. A biological unit image composed of a single hFasLECD trimer (yellow) and a triply bound hDcR3 monomer (white) is depicted as space filling models. The N-terminal residues of hFasLECD subunits in this model are shown in green. Left panel, a horizontal view. One of the position of N-terminal tag sequence attachment sites is arrowed. Right panel, a vertical view. The structure was drawn using the atomic coordinates (ID: 4smv) and the graphic software (jV) provided by Protein Data Bank Japan (PDBj). c) SDS-PAGE analysis of initial stepwise salt-gradient fractionation of the materials in *P. pastoris* culture medium using a cation-exchange column (Hi-Trap S 5 ml). Basal buffer: 50 mM sodium acetate (pH 5.5). Lanes: M, Molecular-weight size markers; 1, before fractionation; 2, flow-through fraction; 3, 0 mM NaCl fraction; 4, 50 mM NaCl fraction; 5, 300 mM NaCl fraction; 6, 500 mM NaCl fraction. AOX-1: *P. pastoris* alcohol oxidase 1, hFasLECD dimer: disulfide-bridged dimer of hFasLECD subunits, hFasLECD monomer: monomeric hFasLECD subunit. (PPTX 333 kb)

Additional file 2: High-performance size-exclusion chromatography profile of hFasRECD-Fc. Absorbance at 280 nm (blue) and 550 nm (red) was used for the detection. (PPTX 88 kb)

Additional file 3: Preparation of rFab’-MTZ. a) SDS-PAGE analysis of pepsin digestion of whole rabbit IgG. Lanes: M, molecular-weight size markers; 1, before digestion; 2, after digestion. b) Fractionation by high-performance size-exclusion chromatography. Panels: left, rF(ab’)_2_, peak fraction shown in the underbar was collected; right, rFab’-MTZ, peak fraction shown in the underbar was collected. Retention time of each peak is shown. (PPTX 231 kb)

## Abbreviations

hFasLECD: human Fas ligand extracellular domain
hFasRECD: human Fas receptor extracellular domain
hFasRECD-Fc: a fusion protein composed of human Fas receptor extracellular domain and human IgG_1_-Fc domain
MTZ: 6-methyl-1, 2, 4, 5-tetrazine group
NaCl: sodium chloride
SDS-PAGE: sodium dodecyl sulfate polyacrylamide gel-electrophoresis
TCO: *trans*-cyclooctene group
Tris-HCl: tris(hydroxymethyl)aminomethane hydrochloride
