# Supplementary material for: Site-specific chemical conjugation of human Fas ligand extracellular domain using trans-cyclooctene – methyltetrazine reactions
Source: BMC Biotechnol. 2017 Jul 3;17:56. doi: 10.1186/s12896-017-0381-2 (PMC5496246; doi:10.1186/s12896-017-0381-2)
Supplement: Supplementary file 1 — Preparation of NFK3G1CG4-hFasLECD. a) Gene structure of expression unit and detailed tag sequences. In the tag sequences, the introduced three lysine residues and the reactive cysteine residue used for chemical modification with either TCO-PEG3-MAL or MTZ-PEG4-MAL are shown in blue and red, respectively. AOX-1 P, P. pastoris alcohol oxidase 1 promoter region; α-Prepro, Saccharomyces cerevisiae α-factor secretion-signal sequence; Tag, tag sequence; hFasLECD (139–281, N184Q, N250Q), human Fas ligand extracellular domain containing deletion mutation from residue 103 to 138 and double substitution mutation (N184Q and N250Q); AOX-1 TT, P. pastoris alcohol oxidase 1 transcription termination region. b) Three dimensional structure of hFasLECD-hDcR3 complex [26]. A biological unit image composed of a single hFasLECD trimer (yellow) and a triply bound hDcR3 monomer (white) is depicted as space filling models. The N-terminal residues of hFasLECD subunits in this model are shown in green. Left panel, a horizontal view. One of the position of N-terminal tag sequence attachment sites is arrowed. Right panel, a vertical view. The structure was drawn using the atomic coordinates (ID: 4smv) and the graphic software (jV) provided by Protein Data Bank Japan (PDBj). c) SDS-PAGE analysis of initial stepwise salt-gradient fractionation of the materials in P. pastoris culture medium using a cation-exchange column (Hi-Trap S 5 ml). Basal buffer: 50 mM sodium acetate (pH 5.5). Lanes: M, Molecular-weight size markers; 1, before fractionation; 2, flow-through fraction; 3, 0 mM NaCl fraction; 4, 50 mM NaCl fraction; 5, 300 mM NaCl fraction; 6, 500 mM NaCl fraction. AOX-1: P. pastoris alcohol oxidase 1, hFasLECD dimer: disulfide-bridged dimer of hFasLECD subunits, hFasLECD monomer: monomeric hFasLECD subunit. (PPTX 333 kb) [file 12896_2017_381_MOESM1_ESM.pptx]

## Slide 1
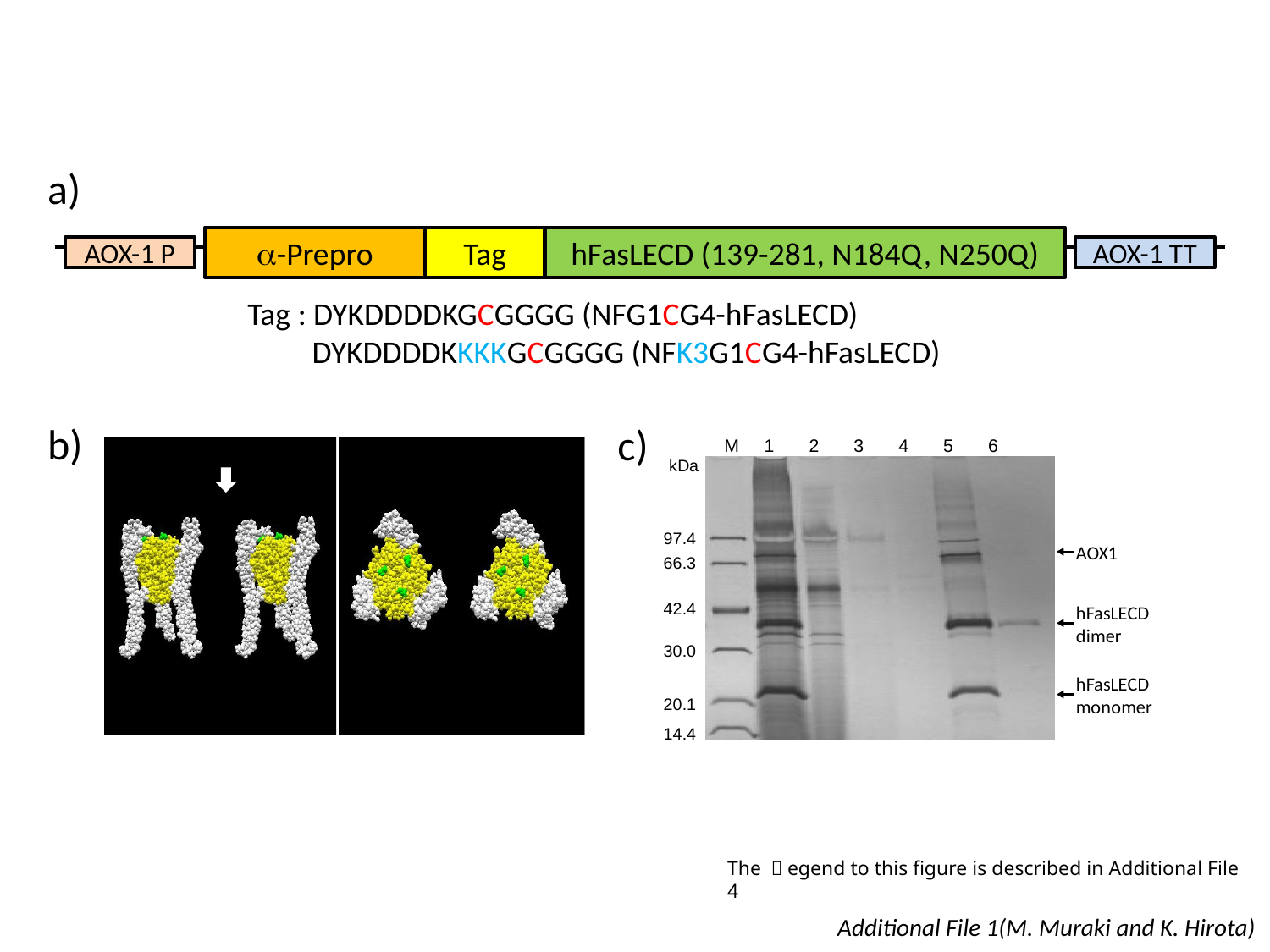

a)
b)
c)
a-Prepro
Tag
hFasLECD (139-281, N184Q, N250Q)
AOX-1 P
AOX-1 TT
Tag : DYKDDDDKGCGGGG (NFG1CG4-hFasLECD)
 DYKDDDDKKKKGCGGGG (NFK3G1CG4-hFasLECD)
The ｌegend to this figure is described in Additional File 4
Additional File 1(M. Muraki and K. Hirota)
