# Supplementary material for: Site-specific chemical conjugation of human Fas ligand extracellular domain using trans-cyclooctene – methyltetrazine reactions
Source: BMC Biotechnol. 2017 Jul 3;17:56. doi: 10.1186/s12896-017-0381-2 (PMC5496246; doi:10.1186/s12896-017-0381-2)
Supplement: Supplementary file 2 — High-performance size-exclusion chromatography profile of hFasRECD-Fc. Absorbance at 280 nm (blue) and 550 nm (red) was used for the detection. (PPTX 88 kb) [file 12896_2017_381_MOESM2_ESM.pptx]

## Slide 1
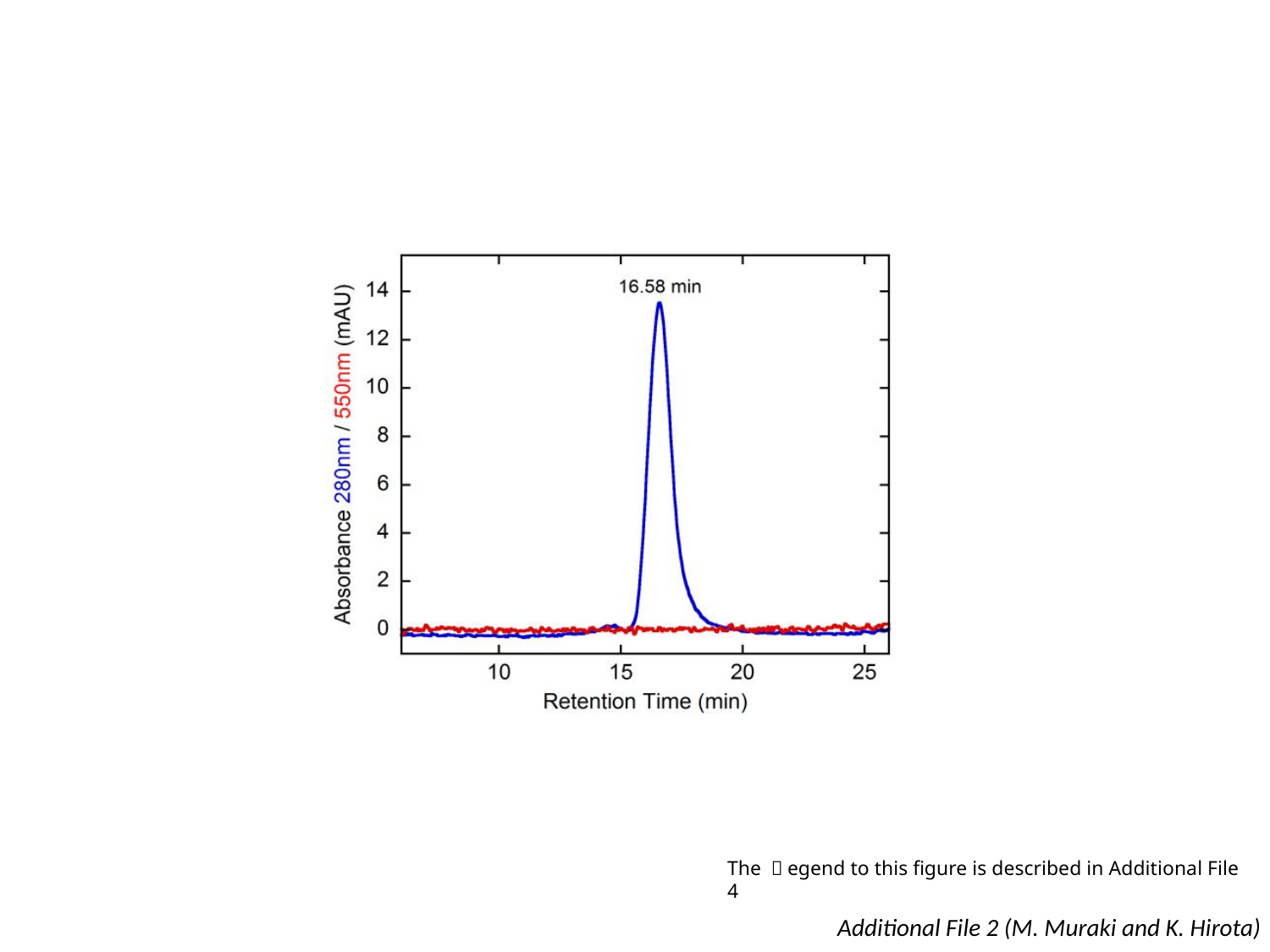

The ｌegend to this figure is described in Additional File 4
Additional File 2 (M. Muraki and K. Hirota)
