# Supplementary material for: Site-specific chemical conjugation of human Fas ligand extracellular domain using trans-cyclooctene – methyltetrazine reactions
Source: BMC Biotechnol. 2017 Jul 3;17:56. doi: 10.1186/s12896-017-0381-2 (PMC5496246; doi:10.1186/s12896-017-0381-2)
Supplement: Supplementary file 3 — Preparation of rFab’-MTZ. a) SDS-PAGE analysis of pepsin digestion of whole rabbit IgG. Lanes: M, molecular-weight size markers; 1, before digestion; 2, after digestion. b) Fractionation by high-performance size-exclusion chromatography. Panels: left, rF(ab’)2, peak fraction shown in the underbar was collected; right, rFab’-MTZ, peak fraction shown in the underbar was collected. Retention time of each peak is shown. (PPTX 231 kb) [file 12896_2017_381_MOESM3_ESM.pptx]

## Slide 1
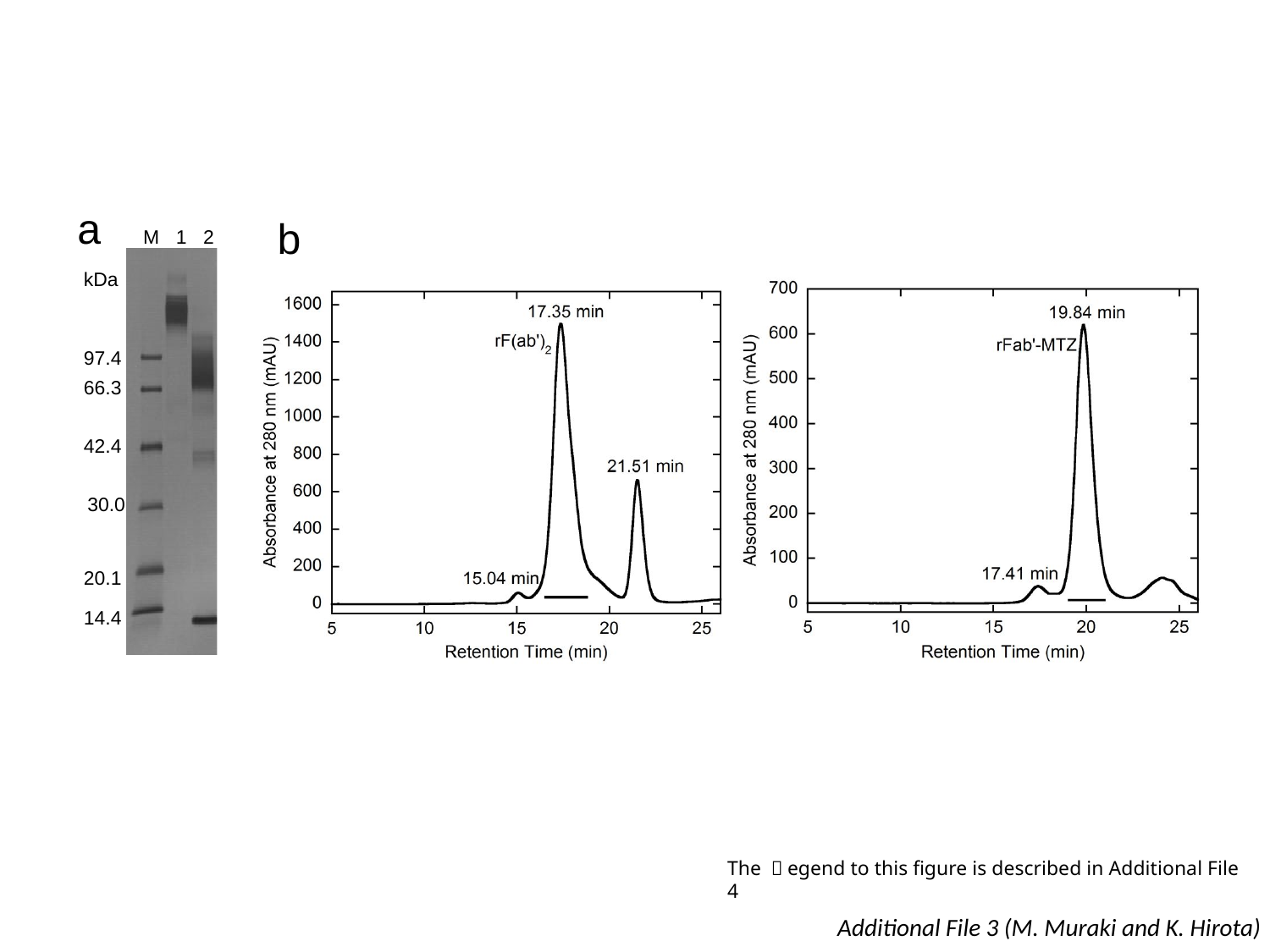

a
 M 1 2
kDa
97.4
66.3
42.4
30.0
20.1
14.4
b
The ｌegend to this figure is described in Additional File 4
Additional File 3 (M. Muraki and K. Hirota)
